# Chondromatose synoviale du poignet révélée par un syndrome de canal carpien, un cas exceptionnel

**DOI:** 10.11604/pamj.2016.23.6.8690

**Published:** 2016-01-20

**Authors:** Younes Mhammdi, Ahmed El Bardouni

**Affiliations:** 1 Service de Traumatologie Orthopédie, Hopital Ibn Sina, CHU Rabat, Maroc

**Keywords:** Chondromatose synoviale, poignet, canal carpien, Synovial chondromatosis, wrist, carpal tunnel

## Image en médecine

Les auteurs rapportent le cas d'une patiente de 53 ans atteinte de cette pathologie qui s'est révélée par un syndrome de canal carpien. L'image montre du coté droit une vue opératoire après ouverture du canal carpien montrant le nerf médian écarté faisant apparaître la chondromatose, à gauche le plus gros chondrome après extraction.

**Figure 1:**
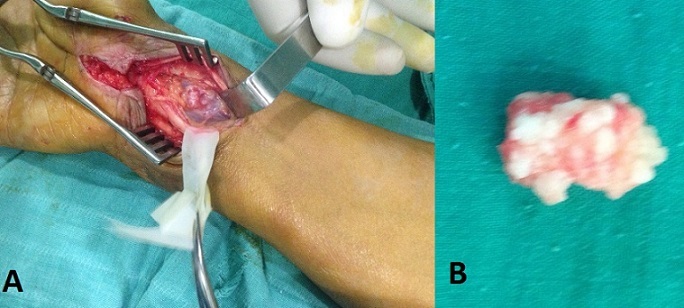
(A) le canal carpien après ouverture du rétinaculum des fléchisseurs, le nerf médian écarté et mis sur lacs faisant apparaître un chondrome; (B) le plus grand chondrome après extraction

